# The Diagnostic and Prognostic Values of C-Reactive Protein and Procalcitonin during Bacterial Infections in Decompensated Cirrhosis

**DOI:** 10.1155/2018/5915947

**Published:** 2018-12-30

**Authors:** Sana Khedher, Nasreddine Fouthaili, Amira Maoui, Sirine Lahiani, Mohamed Salem, Kahena Bouzid

**Affiliations:** ^1^Intensive Care Unit-Hepato and Gastroenterology Department, Charles Nicolle Hospital, Tunis, Tunisia; ^2^Faculty of Medicine, University of Tunis El Manar, El Manar, Tunisia; ^3^Laboratory of Clinical Biochemistry, Charles Nicolle Hospital, Tunis, Tunisia; ^4^LR18ES03, Laboratory of Neurophysiology, Cellular Physiopathology and Biomolecules Valorisation, University of Tunis, El Manar 2092, Tunis, Tunisia

## Abstract

**Background:**

Bacterial infection (BI) represents the main cause of decompensation and death in cirrhotic patients. Procalcitonin (PCT) and C-reactive protein (CRP) are two widely used biomarkers that may be helpful for early detection of BI especially in the presence of inflammation. Their accuracy for the diagnosis of BI in patients with chronic liver disease has been a subject of debate. In this study, we aimed to learn whether PCT and CRP would be helpful as early markers of BI in patients with cirrhosis and to evaluate their prognostic value in terms of mortality.

**Subjects and Methods:**

We retrospectively included 92 adult patients with decompensated cirrhosis. PCT and CRP plasma levels were obtained within the first 24 hours of admission. Their diagnostic and prognostic values were compared using the appropriate statistical analysis.

**Results:**

Ninety-two patients were included. BI was diagnosed in 60 patients (65%). Mean white blood cell (WBC) count (*p* = 0.005) and PCT and CRP serum levels (*p* < 0.001) were higher in the BI group than in the non-BI (NBI) group. The diagnostic accuracy of CRP and PCT for the diagnosis of BI was better than that of WBC. CRP was the most sensitive marker (70%) while PCT was the more specific (96.6%). No one of those biomarkers was predictive of 3-month mortality in patients with BI.

**Conclusion:**

Regarding BI in patients with decompensated cirrhosis, CRP maintains efficiency slightly higher than that of the PCT without being discriminative. However, no prognostic value has been established for these markers.

## 1. Introduction

Cirrhosis is the advanced stage of a liver condition due to a chronic inflammatory and fibrosing prolonged evolving process. Cirrhotic patients are more susceptible to bacterial infection (BI) because of humeral and cell-mediated immunodeficiency, splanchnic ganglia colonization, bacterial translocation phenomena, and ventilatory disorders related to encephalopathy and ascites [1, 2]. BI is diagnosed in 30 to 50% of admitted cirrhotic versus 5 to 7% in noncirrhotic patients [3]. The diagnosis of BI in patients with cirrhosis is made difficult by some clinical and biological abnormalities commonly observed in those patients such as liver dysfunction, inflammation, chronic hypersplenism, abdominal distension, and neurological disorders in addition to the frequent use of betablockers. Despite the great improvement of BI management in cirrhotics, the mortality is still high. An early diagnosis and treatment of this condition, using biomarkers such as procalcitonin (PCT) and C-reactive protein (CRP), can contribute to reduce this mortality. This work is aimed at evaluating and comparing diagnosis and prognosis performance of CRP and PCT during BI in patients admitted for decompensated cirrhosis.

## 2. Methods

This 14-month observational retrospective study was conducted in the Hepato-gastroenterology Department of the Charles Nicolle University Hospital of Tunis from 01 September 2015 to 30 October 2016. We included patients admitted for decompensate cirrhosis. Cirrhosis was diagnosed according to histological and/or clinical, biological, and ultrasound findings suggesting portal hypertension and hepatocellular insufficiency. Decompensated cirrhosis was defined by the presence of ascites, jaundice, variceal hemorrhage, or hepatic encephalopathy. Patients not included in the study were those who were receiving intravenous antibiotics and those who were suffering from a systemic disease or a cancer including hepatocellular carcinoma. CRP and PCT are part of the routine biological sample performed at admission; we did not have to get patients' consent. The following severity and prognostic scores were used in our study: APACHE II score [4], Child-Turcotte-Pugh (CP) criteria [5], Model for End-Stage Liver Disease score (MELD) [6], CLIF-sequential organ failure assessment score (CLIF-SOFA), and Acute-on-chronic liver failure (ACLF) [7]. Blood samples for biological marker analysis as well as microbiological samples were obtained on admission. Serum CRP levels were measured using direct immunoturbidimetry on the Architect C 8000 Chemistry System (Abbott Diagnostics, USA). Enzyme-linked fluorescent immunoassay (ELFA) on mini-VIDAS (bioMérieux, France) was used for PCT tests. The patients were divided into a BI group and a non-BI (NBI) group. Dichotomous variables were expressed as percentages and continuous variables as mean ± standard deviation. Statistical comparisons were performed using, respectively, Student's *t*-test and *χ*^2^. Analysis of receiver operating characteristic (ROC) curves was used to determine diagnostic accuracy, sensitivity, and specificity and the Youden index to determine suggested cutoff. All analyses were conducted by SPSS 20.0 (SPSS Inc., Chicago, IL, USA). A *p* value of <0.05 was considered significant. Three-month mortality risk factors were determined by the application of multivariate logistic regression analysis. The authors state the absence of conflict of interest.

## 3. Results

Ninety-two patients were included with a sex ratio of 0.96 and a mean age of 63 ± 13 years (19-91). Mean follow-up time was 4.5 ± 4 years (3 months-26 years). Fifty-seven patients (61%) were CP class B and 32% class C. Hepatitis C was the most common etiology of cirrhosis (42%). They were mainly admitted for edemato-ascitic decompensation and the main source of infection was pulmonary tract infection (62%) and spontaneous bacterial peritonitis (23%). Baseline characteristics, cirrhosis etiologies, causes for admission, infection sources, and severity scores are summarized in Table 1. Distributions of sex, age, and comorbidities were similar between the two groups (Table 2). BI group had significantly higher encephalopathy rate (*p* = 0.005), higher body temperature (*p* = 0.008), higher heart rate (*p* = 0.025), higher total serum bilirubin level (*p* = 0.021), higher international normalized ratio (*p* = 0.005), lower prothrombin time (*p* = 0.005), and higher MELD score (*p* < 0.01). Mean serum levels of CRP (*p* < 0.001), PCT (*p* < 0.001), and white blood cells (WBC) (*p* = 0.002) were significantly higher in the BI group than in the NBI group (Table 2). The results of the logistic regression analysis showed that only MELD score > 15, encephalopathy, and PCT ≥ 0.5were significantly correlated to BI (Table 3). We evaluated the effectiveness of WBC, PCT, and CRP measurements in predicting infection in decompensated cirrhosis using the assessment of ROC curves. The area under the curve (AUC), sensitivity, specificity, positive and negative predictive values, and suggested cutoff values for each marker are summarized in Table 4. ROC analysis of CRP serum level showed the slightly higher AUC (0.745). PCT was more specific (96.6% vs. 75%) whereas CRP was more sensitive (70 vs. 45%).

### 3.1. Three-Month Mortality

**Figure 1:**
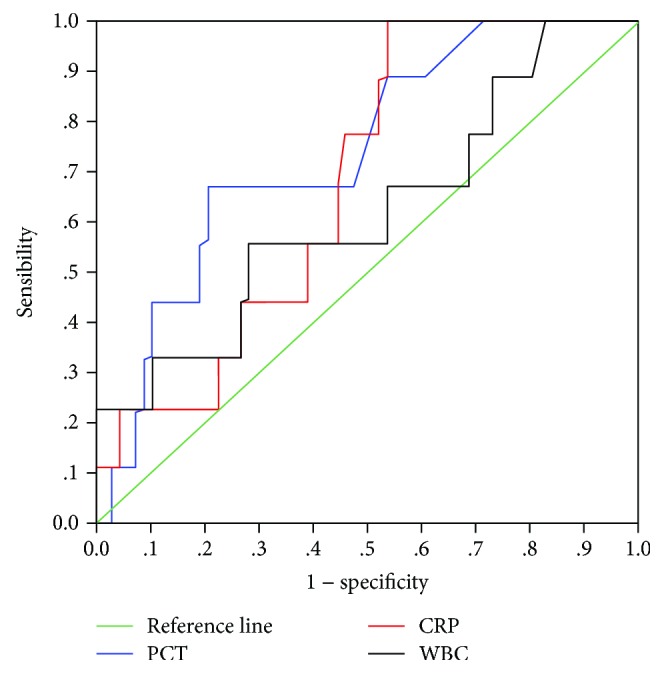
ROC curves for procalcitonin (PCT), C-reactive protein (CRP), and white blood cells (WBC) in predicting 3-month mortality.

Three-month mortality rate was 27%. Patients in the BI group had higher 3-month mortality rate (34% vs. 3%, *p* = 0.008) than those in the NBI group. However, multivariate risk factor analysis indicated that BI was not an independent 3-month mortality risk factor (Table 5). PCT serum level was significantly associated with 3-month mortality (*p* < 0.001). ROC analysis of PCT serum level showed the AUC of 0.735 (CI 95%: 0.576-0.895). Suggested cutoff was 0.5 ng/mL; sensitivity, specificity, positive predictive value, and negative predictive value were, respectively, 66.7%, 77.6%, 59%, and 82% (Figure 1). In the BI group, deceased patients were more often Child-Pugh class C and had lower temperature on admission, higher Child-Pugh score, higher leukocyte, neutrophil, and platelet counts, lower prothrombin time, and higher serum CRP, PCT, and creatinine levels (Table 6). In multivariate logistic regression mode, CP class C, lower temperature on admission, higher CP score, and higher platelet count were retained as predictors' parameters for three-month mortality (Table 7).

## 4. Discussion

In our study, the high rate of BI among patients with decompensated cirrhosis can be explained by the advanced liver failure observed in these patients. Our results showed that PCT and CRP serum levels were significantly higher in the BI group, and thus, these markers were useful in the diagnosis of BI in decompensated cirrhosis. Although they are mainly produced by the liver, hepatic insufficiency does not affect their diagnostic accuracy [8]. That is explained by a maintained hepatic production [9–11] and/or an extrahepatic production [12–15] of those two biomarkers during cirrhosis. On the other side, WBC count is deeply influenced by hypersplenism even if their production may be maintained. That can explain the less BI diagnosis accuracy of WBC observed in our study. Several studies have shown the reliability of PCT and CRP for the diagnosis of infection during cirrhosis with important AUC. In a recent meta-analysis including 1144 patients with liver cirrhosis, AUC for PCT and CRP was, respectively, 0.92 (95% CI: 0.89-0.94) and 0.87 (95% Cl: 0.84-0.90) [16]. This is probably due to differences in demographic characteristics and cirrhosis severity observed in our population. The cutoffs used are also different from ours. In the present study, diagnostic accuracy of CRP and PCT was comparable but it is still relatively low. That is why we think that PCT and CRP cannot be used alone to either confirm or exclude BI diagnosis. We found that, for the diagnosis of BI, CRP was more sensitive (70% vs. 45%) while PCT was more specific (96.9% vs. 75%). Similar results were reported by Lin et al.: CRP sensitivity was higher (87% vs. 79%) while PCT was more specific (89% vs. 85%) [16]. In addition, the CRP had a more discriminative value in the Papp study [17]. In order to enhance the diagnostic performance of CRP and PCT, the use of an algorithm that integrates clinical data with PCT or CRP can be helpful. In addition, it may be interesting to study the usefulness of an algorithm combing CRP and PCT.

### 4.1. Mortality

We have reconfirmed the gravity of BI in cirrhotics as already attested [18]. Three-month mortality was twelve times higher in infected patients compared to noninfected patients (34% vs. 3%, *p* = 0.08). However, multivariate risk factor analysis indicated that BI was not an independent 3-month mortality risk factor. Lazzarotto et al. did not find any association between infection at admission and death before the seventh day of hospitalization [19]. In Khot et al. study, sepsis was not objectively an independent predictor of mortality [20]. In our study, only serum PCT level was significantly related to 3-month mortality. The relationship between PCT and CRP levels and mortality reported in previous studies was contradictory [19, 21–23]. However, in the BI group, PCT and CRP did not show any prognostic value. That can be explained by the high severity of cirrhosis in the NBI group. In fact, the CP score influenced death.

Our study has limitations. It is a monocentric study with a relatively small number of patients. In addition, PCT measurement was performed on admission regardless of the presence or the absence of infection.

## 5. Conclusion

Regarding BI in patients with decompensated cirrhosis, CRP maintains efficiency slightly higher than that of the PCT without being discriminative. However, no prognostic value has been established for these markers. In order to improve the diagnosis and reliability of prognosis, it may be interesting to combine them with each other or with other biological and/or clinical parameters.

## Figures and Tables

**Table 1:** Baseline characteristics of patients.

*Number of patients*	92
*Mean age, years*	63 ± 13 (19-91)
*Male,n*	45
*Female,n*	47
*Cirrhosis etiology,n(%)*	
Hepatitis C	39 (42)
Hepatitis B	9 (10)
Autoimmune hepatitis	7 (8)
Primary biliary cirrhosis	8 (9)
Alcohol	3 (4)
Nonalcoholic steatohepatitis	7 (8)
Cryptogenic	13 (14)
Others	4 (5)
*Causes for admission,n(%)*	
Edemato-ascitic decompensation	46 (50)
Hepatic encephalopathy	23 (25)
Gastro-intestinal bleeding	14 (15)
Fever	14 (15)
Others	9 (10)
*Infection source,n(%)*	
Pulmonary tract infection	37 (62)
Urinary tract infection	12 (20)
Spontaneous bacterial peritonitis	14 (23)
Skin infection	5 (8)
Multiple sites	6 (10)
Bacteremia	9 (14)
Isolated	3 (5)
Associated to another source	6 (10)
*Severity scores*	
Child-Pugh class (%)	
A	7
B	61
C	32
*Child-Pugh, mean*	10 ± 2 (6–15)
*ACLF (%)*	
Grade 1	33
Grade 2	32
Grade 3	35
*MELD score, mean*	18 ± 7 (8-35)
*APACHE II score, mean*	20 ± 5 (12-34)

MELD: Model for End-Stage Liver Disease; APACHE II: Acute Physiology and Chronic Health Disease Classification System II; ACLF: Acute-on-chronic liver failure.

**Table 2:** Clinical and laboratory variables associated with infection on admission.

	Bacterial infection group *n* = 60	Nonbacterial infection group *n* = 32	*p* value
Age (yr)	60 ± 12	64 ± 15	0.509
Male/female	30/30	15/17	0.775
Medical history			
Diabetes mellitus	38%	44%	0.614
Hypertension	28%	41%	0.231
Tabagism	28%	19%	0.350
Alcoholism	15%	13%	0.786
Child-Pugh (class)			
A	1%	6%	0.123
B	28%	44%	0.256
C	70%	50%	0.456
MELD score	20 ± 7	15 ± 5	<.001
APACHE II score	13 ± 8	15 ± 6	0.855
CLIF-SOFA score	15 ± 6	16 ± 6	0.757
ACLF			
Grade 1	32%	38%	0.573
Grade 2	33%	28%	0.609
Grade 3	34%	34%	0.952
Duration of stay (d)	12 ± 8	12 ± 6	0.766
3-month mortality	34%	3%	0.008
Vital signs			
Body temperature (°C)	38 ± 0.7	37 ± 0.5	0.008
Heart rate (beats/min)	97 ± 15	85 ± 18	0.025
Respiratory rate (breaths/min)	21 ± 4	20 ± 5	0.189
Systolic blood pressure (mm Hg)	108 ± 21	111 ± 20	0.393
Diastolic blood pressure (mm Hg)	62 ± 13	66 ± 10	0.193
Encephalopathy	72%	37%	0.005
Ascite	85%	84%	0.647
WBCs (cells/lL)	8750 ± 5005	5672 ± 2734	0.002
Neutrophil (cells/lL)	6430 ± 4456	3668 ± 2151	0.001
Platelet (cells/lL)	105056 ± 80901	91000 ± 42063	0.361
Serum BUN (mmol/L)	8.8 ± 5.2	7.4 ± 5.9	0.282
Serum creatinine (*μ*mol/L)	116 ± 101	90 ± 51	0.167
AST (IU/L)	78 ± 106	56 ± 38	0.350
ALT (IU/L)	43 ± 55	40 ± 55	0.803
Total bilirubin (mg/L)	102 ± 137	43 ± 45	0.021
PT (%)	48 ± 18	58 ± 16	0.007
INR	1.91 ± 0.64	1.55 ± 0.36	0.005
Albumin (g/L)	25 ± 5	26 ± 5	0.579
Serum sodium	132 ± 5	134 ± 4	0.177
CRP (mg/L)	46 ± 44.5	19.61 ± 24.95	<.001
PCT (ng/mL)	1.84 ± 4.07	0.20 ± 0.48	<.001

ALT: alanine aminotransferase; AST: aspartate aminotransferase; CRP: C-reactive protein; PCT: procalcitonin; INR: international normalized ratio; WBC: white blood cell; PT: prothrombin time; MELD: Model for End-Stage Liver Disease; APACHE II: Acute Physiology and Chronic Health Disease Classification System II; BUR: blood urea nitrogen; CLIF-SOFA: chronic liver failure-sequential organ failure assessment; ACLF: Acute-on-chronic liver failure.

**Table 3:** Univariate and multivariate analysis of factors associated with bacterial infection.

	Bacterial infection diagnosis	
	Univariate analysis	Multivariate analysis
Age > 60 years	0.609	0.285
Gender	0.775	0.399
Tabagism	0.35	0.376
Alcoholism	0.786	0.076
Diabetes mellitus	0.614	0.75
Hypertension	0.231	0.535
Duration of cirrhosis	0.170	0.473
Length of hospitalization	0.291	0.843
Child-Pugh score	0.311	0.199
MELD score > 15	0.001	0.022
Ascite	0.937	0.744
Encephalopathy	0.001	0.021
WBC ≥ 6635 cells/lL	0.005	0.22
CRP ≥ 20 mg/L	0.000	0.184
PCT ≥ 0.5 ng/mL	0.000	0.009

WBC: white blood cells; CRP: C-reactive protein; PCT: procalcitonin; MELD: Model for End-Stage Liver Disease.

**Table 4:** The results of ROC curve analysis for bacterial infection diagnosis.

	AUC (95% CI)	*p*	Suggested cutoff	Sensitivity (%)	Specificity (%)	PPV (%)	NPV (%)
*WBC*	0.694 (IC 95%: 0.585-0.803)	**0.002**	**6635**	**65**	**65.5**	**78**	**50**
*CRP*	0.745 (IC 95%: 0.635-0.855)	**<.001**	**20**	**70**	**75**	**84**	**57**
*PCT*	0.741 (IC 95%: 0.639-0.843)	**<.001**	**0.5**	**45**	**96.6**	**97.5**	**59**

WBC: white blood cells. CRP: C-reactive protein; PCT: procalcitonin; PPV: positive predictive value; NPV: negative predictive value.

**Table 5:** Univariate and multivariate analysis of factors associated with 3-month mortality in admitted patients with decompensated liver cirrhosis.

	Univariate analysis	Multivariate analysis
Child-Pugh (class)	0.006	0.007
Encephalopathy	0.013	0.005
Infection	0.001	0.485
Heart rate	0.005	0.092
Temperature	0.055	0.547
WBC	0.001	0.331
Neutrophil count	<0.001	0.155
Platelets	<0.001	0.490
PT %	0.029	0.321
INR	0.036	0.962
CRP	0.001	0.053
PCT	0.018	<0.001
Serum creatinine	0.014	<0.001
AST	0.095	<0.001
Child-Pugh (score)	0.009	0.006
MELD	<0.001	0.091

WBC: white blood cells; INR: international normalized ratio; CRP: C-reactive protein; PT: prothrombin time; PCT: procalcitonin; AST: aspartate aminotransferase; MELD: Model for End-Stage Liver Disease.

**Table 6:** Clinical and laboratory variables associated with 3-month mortality in the BI group.

	Surviving	Deceased	*p* value
Sex	16/21	9/14	0.184
Child-Pugh (class)			
A	0%	3%	0.427
B	4%	43%	0.001
C	96%	54%	0.001
Child-Pugh score	10 ± 2	11 ± 1	0.004
Respiratory rate	21 ± 3	21 ± 4	0.720
Temperature	38 ± 0.8	37 ± 0.6	0.004
WBC (cells/lL)	7632 ± 3651	10549 ± 6314	0.027
Neutrophil count (cells/lL)	5369 ± 3049	8137 ± 5771	0.018
Platelets (cells/lL)	83659 ± 66387	139478 ± 91280	0.008
PT (%)	51 ± 19	41 ± 13	0.031
CRP (mg/L)	37 ± 29	61 ± 60	0.041
PCT (ng/mL)	1.21 ± 2 ± 88	2.84 ± 5.39	0.131
Creatinine (*μ*mol/L)	96 ± 63	150 ± 139	0.045
AST (IU/L)	63 ± 44	91 ± 162	0.396
MELD	18 ± 7	23 ± 6	0.005

PT: prothrombin time; WBC: white blood cells; CRP: C-reactive protein; PCT: procalcitonin; AST: aspartate aminotransferase; MELD: Model for End-Stage Liver Disease.

**Table 7:** Univariate and multivariate analysis of factors associated with 3-month mortality in the BI group.

Variable	Univariate analysis	Multivariate analysis
Sex	0.184	0.900
Child-Pugh (class)		
B	<0.001	0.887
C	<0.001	<0.001
Child-Pugh (score)	0.004	0.627
Respiratory rate	0.720	0.111
Temperature	0.004	0.01
WBC	0.027	0.131
Neutrophil count	0.018	0.075
Platelets	0.008	0.018
Prothrombin time	0.031	0.874
CRP	0.041	0.225
PCT	0.131	0.318
Serum creatinine	0.045	0.197
AST	0.396	0.300
MELD score	0.005	0.411

WBC: white blood cells; CRP: C-reactive protein; PCT: procalcitonin; AST: aspartate aminotransferase; MELD: Model for End-Stage Liver Disease.

## Data Availability

The data used to support the findings of this study are available from the corresponding author upon request.

## Abbreviations

ACLF: Acute-on-chronic liver failure
ALL: Alanine aminotransferase
APACHE II: Acute Physiology and Chronic Health Disease Classification System II
AST: Aspartate aminotransferase
BI: Bacterial infection (group)
CP: Child-Turcotte-Pugh
CLIF-SOFA: Chronic liver failure-sequential organ failure assessment
CRP: C-reactive protein
INR: International normalized ratio
MELD: Model for End-Stage Liver Disease
NBI: Nonbacterial infection (group)
NPV: Negative predictive value
PCT: Procalcitonin
PPV: Positive predictive value
PT: Prothrombin time
WBC: White blood cells.
